# SARS-CoV-2-Antikörper-Antwort auf die zweite COVID-19-Impfung bei neuromuskulären Patienten unter immunmodulierender Therapie

**DOI:** 10.1007/s00115-022-01363-6

**Published:** 2022-08-23

**Authors:** S. S. Justus Hosseini, Anna Dudakova, Karsten Kummer, Jana Zschüntzsch

**Affiliations:** 1 Neuromuskuläres Zentrum Göttingen, Klinik für Neurologie, Universitätsmedizin Göttingen, Robert-Koch-Str. 40, 37075 Göttingen, Deutschland; 2 Institut für Medizinische Mikrobiologie und Virologie, Universitätsmedizin Göttingen, Göttingen, Deutschland

**Keywords:** Impftiter, Anti-Spike-Protein-Antikörper, Mycophenolatmofetil, Intravenöse Immunglobuline, Vaccination titer, Anti-Spike-Protein-Antibody, Mycophenolatmofetil, Intravenous immunoglobulin

## Abstract

Einer erfolgreichen Impfung (adäquater Anstieg der Anti-S[Spike]-Protein-Antikörper) gegen SARS-CoV‑2 (engl. *severe acute respiratory syndrome coronavirus type 2*) wird ein suffizienter Schutz gegen einen schweren Verlauf von COVID-19 (engl. *coronavirus disease 2019*) zugeschrieben. Bei Patient*innen mit chronisch-inflammatorischen Erkrankungen (engl. *„chronic inflammatory diseases“* [CID]) und Immunsuppression ist der Impferfolg weiterhin im wissenschaftlichen Diskurs. Daher evaluierten wir bei Patient*innen mit einer neuromuskulären Erkrankung (NME), die zu regelmäßigen Infusionen von Immunglobulinen in unserer neurologischen Tagesklinik/Ambulanz vorstellig wurden, 2 Wochen nach vollständiger Immunisierung die Antikörpertiter gegen das S1 (S1-Untereinheit des Spike-Proteins) -Antigen von SARS-CoV‑2. Unsere Daten zeigen, dass Patient*innen mit einer chronischen autoimmunen NME und gleichzeitiger immunsuppressiver bzw. immunmodulierender Therapie nach einer Impfung sowohl mit einem mRNA- als auch mit einem Vektorimpfstoff eine Antikörperantwort aufwiesen. Im Vergleich zu gesunden Proband*innen zeigte sich eine vergleichbare Anzahl an Serokonversionen durch die Impfung. Eine Korrelation zwischen Immunglobulindosierung und Impfantwort sowie Infusionsintervall und Impfantwort ließ sich nicht feststellen. Demgegenüber zeigte jedoch insbesondere die Kombination aus Mycophenolatmofetil (MMF) und Prednisolon eine signifikante Reduktion der spezifischen Antikörpersynthese.

## Abstract (en)

Successful vaccination (adequate elevation of anti-spike protein antibodies) is attributed with sufficient protection against a severe course of coronavirus disease 2019 (COVID-19). For patients with chronic inflammatory diseases (CID) and immunosuppression the success of vaccination is an ongoing scientific discourse. Therefore, we evaluated the antibody titer against the S1 antigen of severe acute respiratory syndrome coronavirus 2 (SARS-CoV-2) 2 weeks after complete immunization in patients with an underlying neuromuscular disease (NMD), who presented to our neurological day clinic and outpatient department for regular infusions of immunoglobulins. The data show that patients with chronic autoimmune NMD and simultaneous immunosuppressive or immune modulating treatment show an antibody response after vaccination with both mRNA and vector vaccines. In comparison to healthy subjects there is a comparable number of seroconversions due to the vaccination. A correlation between immunoglobulin dose and vaccination response could not be found; however, in contrast, there was a significant reduction of specific antibody synthesis, especially for the combination of mycophenolate mofetil (MMF) and prednisolone.

## Einleitung

Im Dezember 2020 erfolgte etwa ein Jahr nach Entdeckung des neuartigen Coronavirus (SARS-CoV-2) die Zulassung des ersten Impfstoffs der Hersteller Pfizer-BioNTech. Zuvor veröffentlichte die Ständige Impfkommission (STIKO) gemeinsam mit der Nationalen Akademie der Wissenschaften (Leopoldina) am 2. November 2020 eine Empfehlung zur Priorisierung der Impfung [42]. Auf diese Empfehlung hin trat am 18. Dezember 2020 die Coronavirus-Impfverordnung (CoronaImpfV) in Kraft. Dies hatte zur Folge, dass Patient*innen mit chronischen neuromuskulären Erkrankungen (NME) aufgrund ihrer Grunderkrankung zumindest in die Prioritätsgruppe III (§ 4 Schutzimpfungen mit erhöhter Priorität, Absatz j) eingestuft werden konnten. Personen der Prioritätsgruppe III konnten in Niedersachsen ab dem 10. Mai 2020 geimpft werden^1^

Eine erfolgreiche Impfung gegen SARS-CoV‑2 bietet einen suffizienten Schutz gegen eine Infektion und schwere Krankheitsverläufe [8]. Insbesondere dadurch, dass Patient*innen mit einer chronisch-inflammatorischen Erkrankung (engl. *„chronic inflammatory disease“* [CID]) und Immunsuppression aus den Phase-III-Studien für die durch die FDA (engl. *U.S. Food and Drug Administration*) und EMA (engl. *European Medicines Agency*) zugelassenen COVID-19-Impfstoffe ausgeschlossen waren, ist in diesem Patientenkollektiv der Impferfolg weiterhin im wissenschaftlichen Diskurs [5, 34, 45].

Ursächlich für einen verminderten Impferfolg wird sowohl eine krankheitsbedingte Dysfunktion des Immunsystems [40] als auch eine therapieassoziierte Immunsuppression diskutiert [15]. Dies spiegelte sich bereits vor der Verfügbarkeit der Impfstoffe in einem erhöhten Risiko für COVID-19 bei Patient*innen mit Autoimmunerkrankungen wider [2]. Darüber hinaus scheint die inzwischen etablierte Anwendung von Immunsuppressiva (Dexamethason, Januskinase(JAK)-Inhibitoren und Interleukin(IL)-6-Inhibitoren) zur Therapie von COVID-19 [1, 22, 46] dazu im Widerspruch zu stehen.

Bisher durchgeführte Studien zeigten bereits, dass Patient*innen mit einer CID und Immunsuppression einen reduzierten Antikörpertiter im Vergleich zu einer gesunden Kontrollkohorte ausbilden [21]. Auch Art und Schwere der Immunsuppression scheinen hier entscheidend. So zeigten lediglich 40 % der organtransplantierten Patient*innen mit Immunsuppression einen messbaren Antikörpertiter [27]. Insbesondere ist von einem negativen Einfluss von Cortison und B‑Zell-depletierender Therapie auszugehen [4, 11, 39].

Über die Wirksamkeit der Impfung bei Patient*innen mit NME, Autoimmunerkrankung oder Immunsuppression gibt es bislang wenig Daten.

In der vorliegenden Studie wurde die Serokonversion nach Grundimmunisierung (bestehend aus 2 Impfungen) mit den bis zum 13.07.2021 zugelassenen Impfstoffen, dem mRNA-Impfstoff BNT162b2 (Comirnaty®; BioNTech Manufacturing GmbH, Mainz, Deutschland), mRNA-1273 (Spikevax®; MODERNA BIOTECH SPAIN S.L., Madrid, Spanien) und dem Vektorimpfstoff ChAdOx1 nCoV-19 (Vaxzevria®; AstraZeneca AB, Södertälje, Schweden) bei Patienten*innen mit NME und immunsupprimierender/-modulierender Therapie untersucht.

Zum Nachweis des Impferfolgs führten wir 2 Wochen nach der zweiten Impfung eine serologische Untersuchung zum Nachweis spezifischer IgG-Antikörper gegen das S1-Antigen von SARS-CoV‑2 durch und bestimmten zusätzlich Antikörpertiter gegen das Nukleokapsid.

## Methoden

Wir boten allen Patient*innen mit einer NME, die zu regelmäßigen Infusionen mit Immunglobulinen in unsere Tagesklinik kamen oder eine Verlaufskontrolle unter immunmodulierender Therapie in unserer neuromuskulären Ambulanz erhielten, eine Bestimmung der Impfantwort im Rahmen einer klinischen Routineuntersuchung nach der zweiten Impfung mit einem der in Deutschland zugelassenen Impfstoffe an. Die zweite Impfung lag dabei 2 Wochen ± 5 Tage zurück. Patient*innen, die eine bekannte SARS-CoV-2-Infektion durchgemacht hatten, wurden nicht in die Studie aufgenommen. Die Daten wurden bis zum 03.08.2021 erhoben. Alle Patient*innen stimmten der Teilnahme an der Studie zu.

Zur Bestimmung der Impfantwort durch Bildung von Anti-Spike-Protein-Antikörpern wurde der Anti-SARS-CoV‑2 QuantiVac ELISA IgG (Euroimmun QuantiVac IgG; Euroimmun, Lübeck, Deutschland) verwendet, dessen relative Einheiten pro Milliliter (RE/ml)/„binding antibody units“ pro Milliliter (BAU/ml) mit der Konzentration an neutralisierenden Antikörpern signifikant korreliert [12]. Diese wiederum spiegeln den Impfschutz wider [29]. Die obere Nachweisgrenze liegt bei 384 BAU/ml. Werte zwischen ≥ 25,6 und < 35,2 BAU/ml wurden als grenzwertig und < 25,6 BAU/ml als negativ klassifiziert [14]. Zum Nachweis einer Immunantwort durch eine natürliche Infektion wurde zusätzlich der Antikörpertiter gegen das Nukleokapsid durch einen VIROTECH SARS-CoV‑2 IgA/IgG ELISA (VIROTECH, Rüsselsheim am Main, Deutschland) bestimmt.

## Ergebnisse

In die Studie konnten 31 Patient*innen eingeschlossen werden (Tab. 1). Zwölf der Patient*innen litten an einer chronisch-inflammatorischen demyelinisierenden Polyradikuloneuropathie (CIDP), 6 an einer Myasthenia gravis (MG), 5 an einer Overlap-Myositis, 2 an einer Einschlusskörpermyositis (engl. „inclusion body myositis“ [IBM]) und je ein Patient an einer nekrotisierenden Myositis, Neuromyotonie, Small-Fiber-Polyneuropathie, Lambert-Eaton-Myasthenie-Syndrom (LEMS) und Lewis-Sumner-Syndrom (MADSAM, engl. *multifocal acquired demyelinating sensory and motor neuropathy*).

**Table 1:** 

Alter	Geschlecht	Erkrankung	Immunglobuline (Gramm (g)/Woche)	Immunsuppression	Impfstoff	Anti-Spike-Antikörper-Titer	Immunglobuline in g pro Monat pro kgKG
50	m	Small-Fiber-PNP	90 g/4 Wochen	–	Vaxzevria®	> 384,00	0,82
84	w	IBM	50 g/4 Wochen	–	Comirnaty®	> 384,00	1,00
70	w	Nekrotisierende Myositis	30 g/12 Wochen	–	Comirnaty®	> 384,00	0,13
58	w	Myasthenia gravis	–	Mycophenolatmofetil, Prednisolon	Comirnaty®	< 3,20	–
53	w	CIDP	160 g/Monate	–	Comirnaty®	> 384,00	2,35
65	m	CIDP	60 g/6 Wochen	Prednisolon	Comirnaty®	> 384,00	0,56
65	w	CIDP	55 g/7 Wochen	–	Comirnaty®	> 384,00	0,36
53	w	Nekrotisierende Myositis	80 g/4 Wochen	Mycophenolatmofetil, Prednisolon	Comirnaty®	12,34	1,31
69	m	CIDP	80 g/6 Wochen	–	Comirnaty®	> 384,00	0,65
38	w	CIDP	28 g/Woche s.c.	–	Spikevax®	> 384,00	1,40
45	w	Lambert-Eaton-Myasthenie-Syndrom	50 g/4 Wochen	–	Comirnaty®	> 384,00	0,51
61	m	Myasthenia gravis	–	Ciclosporin, Prednisolon	Comirnaty®	> 384,00	–
59	w	CIDP	100 g/4 Wochen	–	Comirnaty®	> 384,00	0,70
64	w	Overlap-Myositis	–	Mycophenolatmofetil, Prednisolon	Comirnaty®	8,42	–
80	m	MADSAM	100 g/4 Wochen	–	Comirnaty®	> 384,00	2,08
43	w	Overlap-Myositis	120 g/4 Wochen	Mycophenolatmofetil, Prednisolon	Comirnaty®	5,83	1,29
82	w	Overlap-Myositis	50 g/4 Wochen	–	Comirnaty®	> 384,00	1,02
45	w	Overlap-Myositis	200 g/3 Wochen	–	Comirnaty®	> 384,00	3,29
69	m	CIDP	200 g/5 Wochen	Prednisolon	Comirnaty®	> 384,00	2,13
74	m	CIDP	–	Rozanolixizumab, Azathioprin	Comirnaty®	131,72	–
86	w	CIDP	100 g/6 Wochen	–	Comirnaty®	> 384,00	0,78
71	w	Myasthenia gravis	70 g/8 Wochen	Ciclosporin	Spikevax®	188,85	0,57
78	m	Myasthenia gravis	120 g/6 Wochen	Azathioprin	Spikevax®	> 384,00	0,67
52	m	CIDP	100 g/4 Wochen	Mycophenolatmofetil, Prednisolon	Comirnaty®	> 384,00	0,81
86	m	CIDP	40 g/3 Wochen	Azathioprin, Prednisolon	Spikevax®	> 384,00	0,31
56	w	Overlap-Myositis	–	Azathioprin, Prednisolon	Vaxzevria®/Comirnaty®	> 384,00	–
58	m	Neuromyotonie	–	–	Comirnaty®	> 384,00	–
51	w	Myasthenia gravis	70 g/4 Wochen	–	Comirnaty®	> 384,00	0,88
52	w	Myasthenia gravis	60 g/2 Wochen	–	Vaxzevria®	268,18	1,90
65	m	IBM	60 g/4 Wochen	–	Comirnaty®	> 384,00	0,79
71	w	CIDP	120 g/4 Wochen	–	Vaxzevria®/Comirnaty®	> 384,00	1,33

Dabei verteilte sich das Geschlecht 1:1,5 (Männer zu Frauen; 39 % vs. 61 %). Das mittlere Lebensalter bei Probenentnahme lag bei 63 Jahren. Die ältesten Patient*innen waren ein 86-Jähriger und eine 86-Jährige. Die jüngsten Patientinnen waren zwei 45-jährige Frauen.

25 (81 %) der 31 Patient*innen erhielten eine Immunmodulation mit intravenösen Immunglobulinen. Im Mittel erhielten sie 92,2 g Immunglobuline pro Monat. Auf das Körpergewicht verteilt entspricht dies 1,1 g pro kg Körpergewicht.

Fünf Patient*innen (16 %) erhielten alleinig oder zusätzlich zu Immunglobulinen Mycophenolatmofetil (MMF) mit Prednisolon. Zwei weitere Patient*innen erhielten Azathioprin mit Prednisolon je einmal mit und einmal ohne Immunglobuline. Ein Patient erhielt lediglich Azathioprin mit Immunglobulinen. Ein weiterer Patient erhielt die Kombination aus Azathioprin und Rozanolixizumab. Zwei weitere Patienten erhielten neben Immunglobulinen zusätzlich Prednisolon und 2 weitere Ciclosporin und Prednisolon je einmal mit Immunglobulinen und einmal ohne.

23 der Patient*innen (74 %) wurden 2‑mal im Abstand von 4 bis 6 Wochen mit dem Impfstoff Comirnaty®der Firma Pfizer-BioNTech geimpft. Vier erhielten im selbigen Abstand eine Impfung mit dem Impfstoff Spikevax® der Firma Moderna. Lediglich 2 Patient*innen erhielten eine Impfung mit dem Impfstoff Vaxzevria® von AstraZeneca im Abstand von 9 bis 12 Wochen und weitere 2 Patientinnen erhielten eine Impfung im heterologen Impfschema (AstraZeneca + Pfizer-BioNTech).

30 der 31 Patient*innen (96,77 %) zeigten ein Ansprechen auf die Impfung mit nachweisbaren IgG-Antikörpern gegen das Spike-Protein. 87,1 % erreichten mehr als 35,2 BAU/ml, den Wert, ab dem der ELISA als ausreichend positiv zu werten ist. Bei 24 der 31 Patient*innen (77,4 %) lag dieser oberhalb der oberen Quantifikationsgrenze (384 BAU/ml) des Anti-SARS-CoV‑2 QuantiVac ELISA IgG. Drei der Patient*innen mit einem Anti-Spike-Protein-Antikörpernachweis oberhalb der Quantifikationsgrenze hatten zusätzlich Antikörper gegen weitere Virusbestandteile. Lediglich eine Patientin hatte weder nachweisbare Anti-Spike- noch Anti-Nukleokapsid-Antikörper.

**Figure 1:**
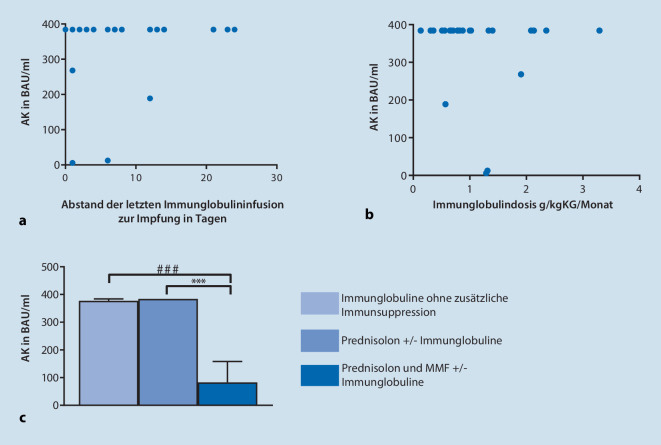

Eine Berechnung des Rangkorrelationskoeffizienten nach Spearman zeigte keine signifikante Korrelation zwischen dem Abstand der letzten Immunglobulininfusion und dem Impferfolg und keine Korrelation zwischen der Immunglobulindosis und dem Impferfolg (Abb. 1a,b).

Es zeigte sich jedoch ein signifikanter Unterschied in der „one-way ANOVA“ (*p* < 0,05) zwischen den Kohorten mit MMF und Prednisolon als Immunsuppressivum vs. keine zusätzliche immunsuppressive Medikation zu den Immunglobulinen und zwischen den Kohorten mit Prednisolon vs. MMF und Prednisolon allein oder in Kombination mit einem Nicht-MMF-Immunsuppressivum (Abb. 1c).

## Diskussion

Unsere Daten zeigen, dass Patient*innen mit einer chronischen autoimmunen NME und gleichzeitiger immunsuppressiver bzw. immunmodulierender Therapie nach Impfungen sowohl mit einem mRNA- als auch Vektorimpfstoff Anti-Spike-Protein-Antikörper bildeten. Es zeigte sich im Vergleich zu gesunden Proband*innen eine vergleichbare Anzahl an Serokonversionen durch die Impfung [13, 37]. Eine Korrelation zwischen Immunglobulindosierung und Impfantwort sowie Infusionsintervall und Impfantwort ließ sich nicht feststellen.

Demgegenüber zeigte jedoch insbesondere die Kombination aus MMF und Prednisolon einen signifikanten Einfluss auf die Antikörpertiter. Die verwendeten Prednisolondosen überschritten 10 mg/d nicht, sodass von einem maßgeblichen Effekt von MMF auszugehen ist. MMF wird zu Mycophenolsäure metabolisiert und inhibiert selektiv und reversibel das Enzym Inosinmonophosphatdehydrogenase, was die Proliferation von B- und T‑Zellen hemmt. Zum einen wurde ein reduziertes Impfansprechen für Dialyse- bzw. transplantierte Patient*innen unter MMF-Therapie [43] und zum anderen für Patient*innen mit rheumatologischen und muskuloskeletalen Erkrankungen nachgewiesen [9]. In der rezenten Studie von Connolly et al. blieb die Antikörperantwort sowohl unter Therapie mit MMF als auch unter Rituximab nach der dritten SARS-CoV-2-Impfung weiterhin suboptimal [9]. Dies deckt sich mit anderen Studien, die B‑Zell-depletierende Medikamente wie Rituximab oder Ocrelizumab untersuchten [3, 17, 23, 26]. Auch die JAK-Inhibitoren und Antimetaboliten (z. B. Methotrexat [MTX]) wurden ebenfalls mit abgeschwächten Antikörper- und Neutralisationstitern in Verbindung gebracht [11, 19]. Allerdings konnte kürzlich für MTX und JAK-Inhibitoren gezeigt werden, dass die dritte Impfdosis zu einem suffizienten Antikörpertiter (> 500 U/ml) bei den meisten Patient*innen führte [9].

Bereits im Sommer 2021 wurde noch unabhängig von der Omikron-Variante postuliert, dass der Status „vollimmunisiert“ nach 2 Impfungen insbesondere bei Patient*innen mit Autoimmunerkrankungen nicht immer verlässlich erreicht wird und eine Überprüfung des Immunstatus nach der Impfung als sinnvoll erachtet wurde, um frühzeitig Impfversagerinnen und -versager zu identifizieren und Impfdurchbrüche zu verhindern [38]. Die Notwendigkeit von Auffrisch- bzw. Nachimpfungen sollte auch in Anbetracht der immer wieder auftretenden Verunsicherung gegenüber Impfungen offen mit den Patient*innen kommuniziert werden, zumal der Nutzen der Impfung und somit der Schutz vor COVID-19 die möglichen Komplikationen deutlich übersteigt. Auch wenn unsere Studie nicht darauf ausgelegt war, berichteten die Teilnehmer*innen von keinen relevanten unerwünschten Impfnebenwirkungen innerhalb der ersten 2 Wochen nach der Impfung. Dies deckt sich mit den kürzlich publizierten Ergebnissen des arztbasierten EULAR-Coronavirus-Vaccine(COVAX)-Registers [31].

Im Zusammenhang mit der fehlenden oder unzureichenden humoralen Immunantwort z. B. durch B‑Zell-depletierende Therapie wäre eine Testung der T‑Zell-Antwort (Spike-spezifische CD4+- und CD8+-T-Zellen) sinnvoll [26], da ein ausreichender Impfschutz durch die zelluläre Immunantwort wahrscheinlich ist [3, 6, 11, 18, 32, 36, 44]. Im Hinblick auf die SARS-CoV-2-spezifische T‑Zell-Antwort sei hervorgehoben, dass auch ein Impfschutz gegen die „besorgniserregenden Varianten“ („variants of concern“) von SARS-CoV‑2 nachgewiesen werden konnte [20], was u. a. auch zu der Empfehlung der Auffrisch‑/Booster-Impfung für die Allgemeinbevölkerung geführt hat.

Allerdings ist im Gegensatz zu den Antikörpertests die Bestimmung der T‑Zell-Antwort aufwendiger (Detektion der antigenspezifischen CD4+- und CD8+-T-Zellen und deren Zytokinausschüttung nach einer entsprechenden *In-vitro*-Stimulation) und noch nicht hinreichend verbreitet, sodass diese vorzugsweise in den Fällen einer fehlenden humoralen Antwort eingesetzt werden sollte [19]. Bezüglich der Antikörperbestimmung sollte ebenfalls bedacht werden, dass ein Antikörper-Titer-Cut-off, ab dem ein ausreichender Schutz gegen eine SARS-CoV-2-Infektion angenommen werden kann, bislang nicht definiert ist [13, 25]. Die in der eigenen Studie verwendeten Grenzwerte entsprechen den Angaben des Testherstellers und sind nicht durch klinische infektiologische Studien validiert. Des Weiteren sollte bedacht werden, dass die Quantifizierung der antigenspezifischen Antikörper im Plasma durch die Gesamt-IgG-Antikörper-Bestimmung mittels ELISA keine sichere Auskunft über die Wirksamkeit der entstandenen humoralen Immunantwort gibt. Dazu wäre die Bestimmung funktioneller Antikörper wie neutralisierender Antikörper notwendig.

Aufgrund der Fallzahlen konnte keine Subgruppenanalyse zu den unterschiedlichen Vakzinen oder neuen Therapien erfolgen. Auch für eine alterskorrigierte Analyse der Impfantwort erwies sich die erhobene Stichprobe als zu gering. Daher wäre eine Fortführung des Impfmonitorings mittels Antikörpertiter wünschenswert, um altersabhängige und therapiebedingte Effekte auf die Impfantwort näher zu bestimmen.

Auch wenn es nur minimale oder keine Wechselwirkungen zwischen Blutprodukten oder Immunglobulinzubereitungen und inaktivierten Impfstoffen gibt (https://www.canada.ca/en/public-health/services/publications/healthy-living/canadian-immunization-guide-part-1-key-immunization-information/page-11-blood-products-human-immune-globulin-timing-immunization.html), wurde initial für die Gabe von intravenösen Immunglobulinen ein zeitlicher Abstand von ca. 1–2 Wochen vor und nach Impfung bzw. eine Halbzeitgabe zwischen den Infusionen von einigen Experten empfohlen [24]. Diese Empfehlung wurde u. a. auf der Basis der bisher bekannten Wirkmechanismen der intravenösen Immunglobuline ausgesprochen. Diese Wirkmechanismen umfassen u. a. die Hemmung des Komplementsystems [16, 41], die Modulation des antiidiotypischen Netzwerks und die Modulation von B‑Zellen und deren Antikörperproduktion [10, 35]. Die phagozytische Aktivität von Makrophagen wird über die Hochregulation des inhibitorischen Fc-Rezeptors beeinflusst, und T‑Zellen werden über die IgG-Bindung an T‑Zell-Rezeptoren, kostimulatorische Moleküle und das fas-/fas-Liganden-System moduliert [7, 10, 28]. Ein weiterer entscheidender Mechanismus ist die Neutralisierung von Zytokinen und eine Regulation von Chemokinen [30, 33].

Zusammenfassend weisen unsere Daten darauf hin, dass die Dosierung der intravenösen Immunglobuline bzw. das Infusionsintervall keinen signifikanten Einfluss auf den Impferfolg der Patient*innen mit verschiedenen NME hat. Demgegenüber sollten bei klassischen Immunsuppressiva und insbesondere bei der Kombination aus MMF und Prednisolon eine Kontrolle der Impftiter erfolgen und vor einer medikamentösen Neueinstellung mit diesen Medikamenten, soweit möglich, der Impfstatus komplettiert werden.

## Footnotes

1 Nds. Ministerium für Soziales, Gesundheit und Gleichstellung Niedersachsen legt Fahrplan für Terminvergabe an Angehörige der Prioritätsgruppe 3 vor. Impfberechtigung erfolgt in drei Stufen beginnend ab 10. Mai.
